# P06 Effectiveness of 16S PCR testing of explanted cardiac devices/tissue (ECDT) samples in the management of device-related infective endocarditis

**DOI:** 10.1093/jacamr/dlaf118.013

**Published:** 2025-07-14

**Authors:** M A N Manchanayaka, C B Owen, J Coldwell, R Palmer, V Sivaprakasam

**Affiliations:** Department of Microbiology, Blackpool Victoria Hospital, Blackpool, UK; Department of Microbiology, Blackpool Victoria Hospital, Blackpool, UK; Department of Microbiology, Blackpool Victoria Hospital, Blackpool, UK; Department of Microbiology, Blackpool Victoria Hospital, Blackpool, UK; Department of Microbiology, Blackpool Victoria Hospital, Blackpool, UK

## Abstract

**Background:**

The Lancashire Cardiac Suite at Blackpool Victoria Hospital (BVH) is a tertiary cardiac centre for NW England, performing 700 CABG, 350 valve replacements and 800 pacemaker insertions annually. Infective endocarditis is a significant aetiological factor amongst these. Recent ESC guidelines recommend a minimum of 6 weeks of antibiotics from the date of a negative blood culture result for infective endocarditis. For patients requiring surgical intervention, this duration is dated from a positive ECDT culture result where available. The Microbiology department at BVH has recently introduced 16S PCR testing for ECDT. If positive, they are treated in the same manner as culture positives.

**Objectives:**

To evaluate the strength of 16S PCR results to support optimization of duration of antibiotics in this patient cohort.

**Methods:**

A descriptive cross-sectional study was performed to analyse 16S PCR results of all ECDT from patients who underwent cardiac surgery for infective endocarditis from January 2021 to December 2023, using data extracted from the Microbiology Laboratory Information Management System (LIMS). These were matched with pre-op blood culture results. Patients were subsequently followed up to December 2024, to identify re-infections, based on information from LIMS and EPS.

**Results:**

A total of 161 patients had ECDT tested by 16S PCR and 111 (69.8%) were positive. Of those, 59 (53.1%) were valve tissues and 52 (46.9%) were pacing leads. Of the 161 patients, 89 (55%) had pre-op blood cultures taken, amongst whom only 22 (24.7%) had positive results. Amongst these, 14 (63%) concurred with 16S PCR results. The remaining were either negative [7 (32%)] or discordant [1 (5%)]. Among 67 patients with negative blood cultures, 50 (74.6%) were positive for 16S PCR, with 10 (20%) *Staphylococcus epidermidis*, 9 (18%) *Staphylococcus aureus,* 3 (6%) *Staphylococcus lugdunensis,* 5 (5%) *Enterococcus faecalis* and 12 (24%) *Streptococcus* sp., amongst others. Out of 111 patients with positive 16S results who were followed up until December 2024, none were readmitted with a subsequent episode of infective endocarditis.

**Conclusions:**

16S PCR testing of ECDT helps to identify the infective agent in those with negative blood cultures. Besides, this would allow optimal duration of antibiotics post-operatively to prevent re-infection of the prosthetic valves. 16S PCR would also be a valuable diagnostic tool in the management of device-related infective endocarditis such as pacemakers and intra-cardiac devices where cultures are negative.

